# Artificial Intelligence in Prostate MRI: Comparison of an AI-Based Software and an Experienced Radiologist for Detecting Clinically Significant Prostate Cancer

**DOI:** 10.3390/curroncol33030151

**Published:** 2026-03-06

**Authors:** Roberto Castellana, Simona Marzi, Andrea Russo, Maria Consiglia Ferriero, Irene Terrenato, Eugenia Papaleo, Giuseppe Navanteri, Davide Vitale, Giuseppe Pizzi, Antonello Vidiri, Luca Bertini

**Affiliations:** 1 Department of Radiology, IRCCS Regina Elena National Cancer Institute, Via Elio Chianesi 53, 00144 Rome, Italy; 2 Medical Physics Laboratory, IRCCS Regina Elena National Cancer Institute, Via Elio Chianesi 53, 00144 Rome, Italy; 3 Department of Pathology, IRCCS Regina Elena National Cancer Institute, Via Elio Chianesi 53, 00144 Rome, Italy; 4 Department of Urology, IRCCS Regina Elena National Cancer Institute, Via Elio Chianesi 53, 00144 Rome, Italy; 5 Biostatistics-Scientific Direction, IRCCS Regina Elena National Cancer Institute, Via Elio Chianesi 53, 00144 Rome, Italy; 6 Clinical Engineering and Technology and Informatic Systems, IRCCS Regina Elena National Cancer Institute, Via Elio Chianesi 53, 00144 Rome, Italy

**Keywords:** artificial intelligence, AI, prostate cancer, MRI

## Abstract

**Background:** Multiparametric MRI is central to detecting clinically significant prostate cancer (csPCa), but diagnostic accuracy depends on reader experience. Artificial intelligence (AI) tools may support prostate MRI interpretation and reduce inter-reader variability. This study compared the detection rate of a trial, non-commercial version an AI-based software (PAROS) with that of an experienced radiologist. **Methods:** This retrospective single-center study included 150 patients who underwent prostate MRI followed by combined systematic and MRI-targeted transperineal biopsy. MRI examinations were interpreted by an experienced radiologist according to PI-RADS v2.1 and independently analyzed using a precommercial trial version of PAROS operating on biparametric MRI. Histopathology served as the reference standard. Detection rate was evaluated using sensitivity, specificity, and positive and negative likelihood ratios (PLR and NLR) at PI-RADS thresholds ≥3 and ≥4. **Results:** CsPCa was present in 63.3% of patients. At both PI-RADS thresholds, PAROS and the radiologist showed comparable sensitivity and specificity, wuth extremely low NLRs, indicating excellent rule-out capability. PLRs were modest and similar at PI-RADS ≥ 3 (1.26 vs. 1.42) and 1.88 for both at PI-RADS ≥ 4. PAROS detected more lesions, particularly in the transition zone. **Conclusions:** PAROS achieved csPCa detection comparable to an experienced radiologist, supporting its role as a decision-support tool in prostate MRI interpretation.

## Simple Summary

Prostate cancer is one of the most common cancers in men, and magnetic resonance imaging (MRI) plays a key role in identifying tumors that require treatment. However, interpreting prostate MRI requires experience, and results may vary between readers. Artificial intelligence tools have been developed to assist radiologists, but their real clinical value is still being evaluated. In this study, we compared an artificial intelligence–based software with an experienced radiologist in detecting clinically significant prostate cancer on MRI, using biopsy results as reference. We found that the software performed similarly to the expert reader, particularly in ruling out significant cancer when MRI findings were negative. Although the software detected more suspicious areas, it should be considered a support tool rather than a replacement for radiologists. These findings suggest that artificial intelligence could help improve consistency in prostate MRI interpretation and support less-experienced readers, with potential impact on future research and clinical practice.

## 1. Introduction

Prostate cancer (PCa) is the most commonly diagnosed cancer among men in both the European Union and the United States, accounting for approximately 23–29% of all male cancer cases [1,2]. The disease exhibits a wide spectrum of clinical behavior, ranging from indolent, low-risk forms that grow slowly and rarely cause mortality, to aggressive, clinical significant forms (csPCa) that can progress to advanced stages and lead to significant cancer-related deaths [3,4].

Multi-parametric MRI (mpMRI) has become an essential tool in the diagnostic pathway for PCa. Its role is emphasized in the latest guidelines from the European Association of Urology (EAU) and the American Urological Association (AUA) [5,6]. MRI is particularly effective in detecting clinically significant PCa (csPCa), which is crucial for guiding treatment decisions. The PRECISION trial, a landmark study, demonstrated that MRI-targeted biopsies detect 12% more csPCa compared to traditional systematic biopsies, while also reducing the diagnosis of indolent, low-risk cancers by 13%. Importantly, the trial showed a 28% reduction in unnecessary biopsies, as men with negative MRI findings could avoid the procedure altogether [7].

To standardize the use of MRI in PCa diagnosis, the Prostate Imaging Reporting and Data System (PI-RADS) was developed. The most recent version, PI-RADS 2.1, provides guidelines for image acquisition, interpretation, and reporting [8]. A meta-analysis of PI-RADS 2.1 found it to have a sensitivity of 96% and a specificity of 43%, with an area under the summary receiver operating characteristic (SROC) curve of 0.86 [9]. Despite these advancements, challenges remain, particularly regarding intra- and inter-reader reproducibility variations [10,11,12,13,14]. Studies have shown that while experienced radiologists miss only 2.6% of csPCa cases, less-experienced readers may fail to detect nearly a third of such cases [15]. According to consensus statements from the European Society of Urogenital Radiology (ESUR) and the European Society of Urological Imaging (ESUI) panel, a radiologist must review at least 400 prostate MRI cases with a minimum of 80% agreement with an expert radiologist to qualify as a basic prostate radiologist [16].

Artificial intelligence (AI), particularly deep learning algorithms, has emerged as a promising solution to these challenges. AI has the potential to streamline the evaluation of MRI images, reduce reporting times, assist less-experienced radiologists in achieving diagnostic accuracy comparable to that of experts, and support second-opinion reviews [17].

Several AI radiology solutions are currently available on the market, offering the potential to reduce the reliance on specialized expertise and decrease inter-reader variability in the evaluation of MRI data. However, limited evidence supports these AI applications’ clinical utility [18,19,20,21,22].

PAROS (Incepto Medical) is a CE-marked, commercially available AI-based tool developed for the automated assessment of prostate MRI scans. The system is designed to identify suspicious lesions and categorize them based on PI-RADS v2.1 criteria, utilizing biparametric T2-weighted and diffusion-weighted imaging (DWI).

This study aimed to compare the detection rate of a precommercial trial version of PAROS software (a pre commercial trial version), released prior to its market launch (July 2024), with that of an experienced radiologist in identifying and grading prostate lesions on MRI, using histopathological findings from systematic and targeted biopsies as the reference standard.

## 2. Materials and Methods

### 2.1. Patients

Institutional Review Board approval was obtained and informed consent was waived given its retrospective design. This single-center retrospective study included patients who underwent combined MRI/US-targeted and systematic biopsies, along with prostate MRI within three months before the biopsy.

All MRI exams and biopsies were conducted between January 2022 and October 2024 for suspected PCa, prompted by either elevated baseline PSA levels or abnormal findings on digital rectal examination, in accordance with clinical practice. Histopathological analysis of the biopsy cores served as the reference standard. Exclusion criteria were: (a) history of csPCa (Gleason score of 7 or higher); (b) patients in active surveillance for nonsignificant PCa; (c) previous transurethral resection of prostate (TURP), radical prostatectomy, radiation therapy or cryoablation (d) inadequate quality of MR images evaluated according to Prostate Imaging Quality (PI-QUAL) v2 score by a radiologist with 4 years of expertise in prostate MRI reporting (R.C.) (e) previous prostate biopsies.

### 2.2. MRI Acquisition Protocol

MRI scans were performed on a 3.0 Tesla MRI (SIGNA Premier, GE Healthcare, Chicago, IL, USA) using a 21-channel surface coil and a PI-RADS v2.1-compliant multiparametric protocol. The protocol included high-resolution T2-weighted imaging in sagittal, coronal, and axial planes, DWI with b-values of 50–800–1000–2000 s/mm^2^, apparent diffusion coefficient (ADC) maps, and dynamic contrast-enhanced sequences following intravenous injection of 0.1 mmol/kg of paramagnetic contrast medium. Diffusion-weighted imaging was acquired using a FOCUS (FOV optimized and constrained undistorted single-shot) sequence, which is known to improve lesion conspicuity by reducing geometric distortions compared to conventional DWI [23]. Details of the MRI protocol are provided in Supplementary Table S1.

### 2.3. MRI Image Interpretation

All MRI exams were evaluated by an experienced radiologist (L.B.) with 25 years of expertise in prostate MRI reporting. Prostate volume was calculated using the ellipsoid formula (anteroposterior diameter × transverse diameter × longitudinal diameter × π/6). Lesions were assessed according to PI-RADS v2.1, and the maximum diameter of the lesion was measured. This evaluation was part of the routine clinical workflow and was blinded to biopsy results. In the per-patient analysis, only the lesion with the highest PI-RADS score was considered, in accordance with PI-RADS recommendations, to ensure one index lesion per patient. In the per-lesion analysis, however, all lesions identified by either the radiologist or the AI tool were included.

### 2.4. AI-Based Software

**Figure 1:**
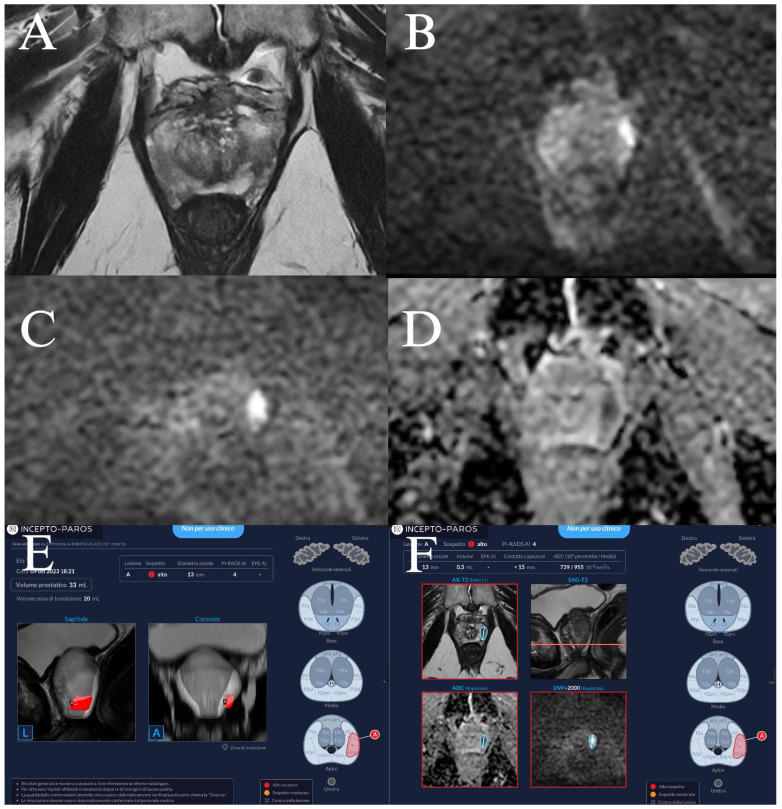
Example of a positive mpMRI prostate nodule in the left peripheral zone of the apex, displaying the original axial T2 (**A**), DWI with a b-value of 1000 (**B**), DWI with a b-value of 2000 (**C**), and ADC map (**D**). The PAROS report consists of a page displaying the measured prostate volume, and all identified lesions (**E**), both in 3D rendering and in axial slices of the prostate (red color is used to indicate PI-RADS 4 and 5 lesions). The next pages of the PAROS report (**F**) present each lesion in the axial T2 sequence, DWI, and ADC map with overlays. In this patient, the biopsy confirmed a csPCa with a Gleason score of 7.

PAROS (Incepto Medical, Paris, France) is a CE-marked, class II-a, commercially available AI tool designed for automated prostate MRI assessment. For this study, we used a trial, non-commercial version of the software released prior to its market launch (July 2024). The deep learning-based algorithm automatically segments the prostate, computes prostate volume, and assigns PI-RADS scores to lesions, aligning with PI-RADS v2.1 guidelines. It should be noted that PAROS operates on biparametric MRI sequences (axial T2-weighted and DWI) and does not utilize DCE data, whereas the radiologist had access to full multiparametric sequences including DCE. This reflects both the AI tool’s design specifications and the current clinical reality that most commercial AI solutions for prostate MRI are trained on biparametric data. The comparison therefore evaluates whether a biparametric AI tool can achieve diagnostic performance comparable to multiparametric expert interpretation—a clinically relevant question given growing interest in reducing gadolinium use and the potential for AI to enable biparametric-only workflows. Dynamic contrast-enhanced sequences are not used by the software. PAROS generates visual and quantitative reports within minutes. An example report is shown in Figure 1.

The PAROS algorithm has not been trained on data from the center conducting this study.

### 2.5. Histopathology

All patients underwent transperineal prostate biopsies in a single session, which included both a systematic 12-core biopsy and an MRI/ultrasound fusion-guided biopsy with 3–4 targeted cores. All procedures were performed using elastic software registration by a urologist with 12 years of clinical experience. Each biopsy core was analyzed by an uropathologist with 25 years of experience. CsPCa was defined as a Gleason score of 7 or higher (ISUP grade group 2 or greater) in any biopsy core, in accordance with the primary endpoint definition used in the landmark PRECISION trial [7].

### 2.6. Statistical Analysis

Descriptive statistics were used to summarize all variables of interest. The AI detection rate was compared to the radiologist and histopathology (gold standard). Sensitivity, specificity, positive and negative likelihood ratios (PLR and NLR) were calculated using two clinically relevant PI-RADS dichotomizations: ≥3 and ≥4. The ≥3 threshold, where PI-RADS 3 lesions are considered test-positive, reflects the standard clinical indication for prostate biopsy and is optimized for high sensitivity (rule-out). The ≥4 threshold, where PI-RADS 3 lesions are considered test-negative, reflects a higher diagnostic certainty for csPCa and is optimized for higher specificity and positive predictive value (rule-in). Analyzing both thresholds provides a comprehensive evaluation of diagnostic performance across different clinical contexts. PLR was defined as the ratio of the probability of a positive test result in people with csPCa to the probability of a positive test result in people without csPCa. NLR was the ratio of the probability of a negative test result in people with csPCa to the probability of a negative test result in people without csPCa. McNemar’s non-parametric test was used to compare differences in sensitivity and specificity between the AI and the radiologist at the same PI-RADS thresholds (≥3 and ≥4). Associations between prostate volume and lesion diameter measurements obtained by PAROS and the radiologist were assessed using the Pearson correlation coefficient to describe the strength of linear association. Systematic differences between measurements were evaluated using the Wilcoxon signed-rank test and comparison of mean values. A *p*-value < 0.05 was considered significant. Analyses used SPSS v.29.0.1 (IBM, Armonk, New York, NY, USA).

## 3. Results

### 3.1. Study Population and Baseline Characteristics

**Figure 2:**
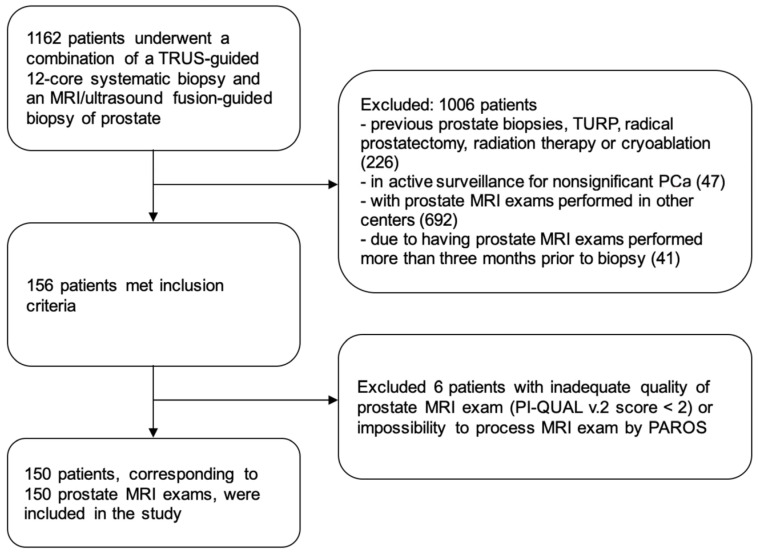
Flow-chart of included patients.

A total of 150 patients were ultimately included in the study, as shown in the schematic diagram of Figure 2. Patients’ characteristics are reported in Table 1.

Most patients (63.3%) had clinically significant prostate cancer on biopsy, and in the majority of cases (71.6%), it was located in the peripheral zone. Because the study’s inclusion criteria required histopathological analysis, only a small number of patients with PI-RADS 1–2 were included. In the per-patient analysis, 134 patients were scored as PI-RADS ≥ 3 by the radiologist (15 PI-RADS 3 and 119 PI-RADS 4–5), and 136 were scored as PI-RADS ≥ 3 by PAROS (15 PI-RADS 3 and 121 PI-RADS 4–5). All MRI examinations met acceptable or optimal quality standards (PI-QUAL v2 score of 2 or 3).

### 3.2. Agreement Between the Radiologist and AI

**Figure 3:**
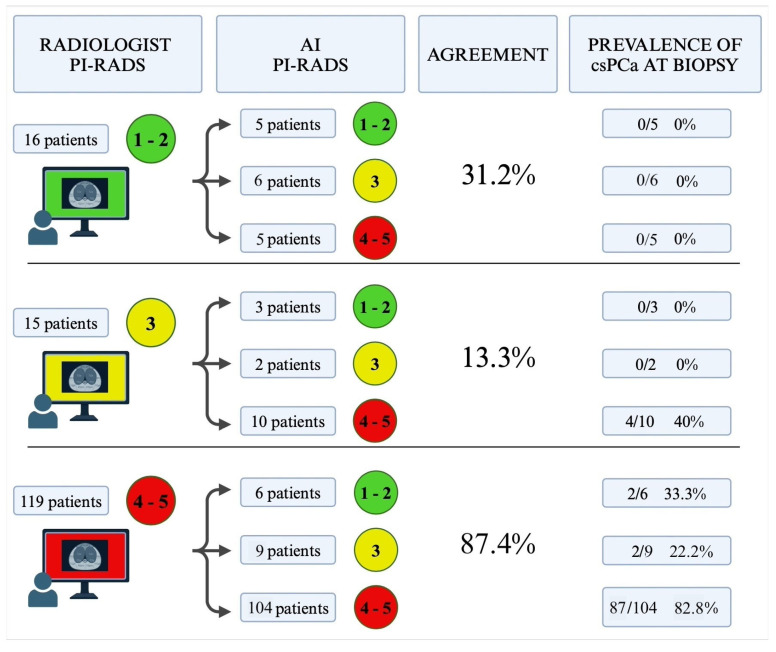
Agreement between the radiologist and the AI-software in classifying PI-RADS lesions and the prevalence of csPCa at biopsy in each subgroup.

In the per-patient analysis, the radiologist agreed with the AI in 31.2% of patients with PI-RADS 1–2, in 13.3% of those with PI-RADS 3, and in 87.4% of those with PI-RADS 4–5 (Figure 3).

In the subgroup of the 104 prostate MRI exams with a PI-RADS lesion of 4 or 5 identified by both the radiologist and the AI, 87 patients (82.8%) were found to have csPCa upon biopsy.

Conversely, in cases where the radiologist assigned a PI-RADS score of 4 or 5 and PAROS assigned a score of 3 (or vice versa), the prevalence of csPCa was lower: 22.2% (2/9) for patients scored as PI-RADS 4 or 5 by the radiologist and 40% (4/10) for those scored as PI-RADS 4 or 5 by the AI system.

Additionally, in nine MRI exams where the radiologist assigned a PI-RADS score ≥3 and PAROS assigned a score <3, only two patients (22.2%) were found to have csPCa upon biopsy.

In the per-lesion analysis, the radiologist detected 169 lesions and the AI software 227 lesions. Of these, 131 lesions were concordantly identified, representing 77.5% of those reported by the radiologist. Among the 96 lesions identified exclusively by PAROS, 57 (59.4%) were located in the transition zone.

### 3.3. Detection Rate of the Radiologist and AI

Table 2 presents the diagnostic accuracy, sensitivity, specificity, PLR and NLR achieved by the radiologist and the AI system in the per-patient analysis for lesions with Gleason scores ≥ 7 across the PIRADS cut-offs of 3 and 4.

**Figure 4:**
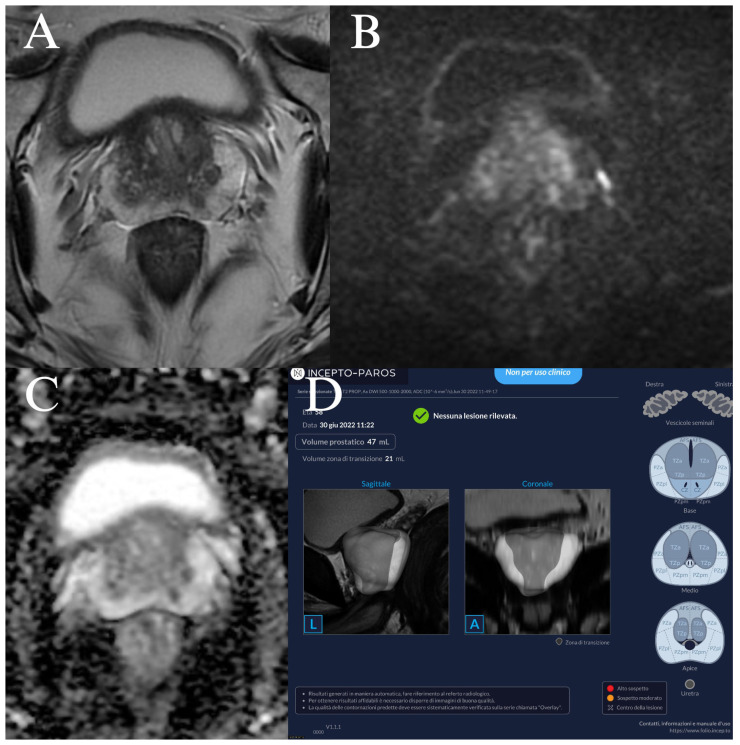
False positive prostate MRI reported by radiologist. Example of a prostate MRI that shows a nodular area of 7 mm in the left peripheral zone of the base which is characterized by hypointensity in T2 (**A**), hyperintensity in DWI (**B**), and hypointensity in ADC map (**C**). The area was scored as PI-RADS 4 by radiologist. The AI-powered tool did not find a lesion with a PI-RADS score of 3 or higher (**D**). At biopsy, no prostate cancer was found. Clinical implication: this case illustrates how false-positive MRI interpretation may lead to unnecessary biopsy, whereas concordant negative AI assessment could potentially support biopsy deferral in selected clinical contexts.

**Figure 5:**
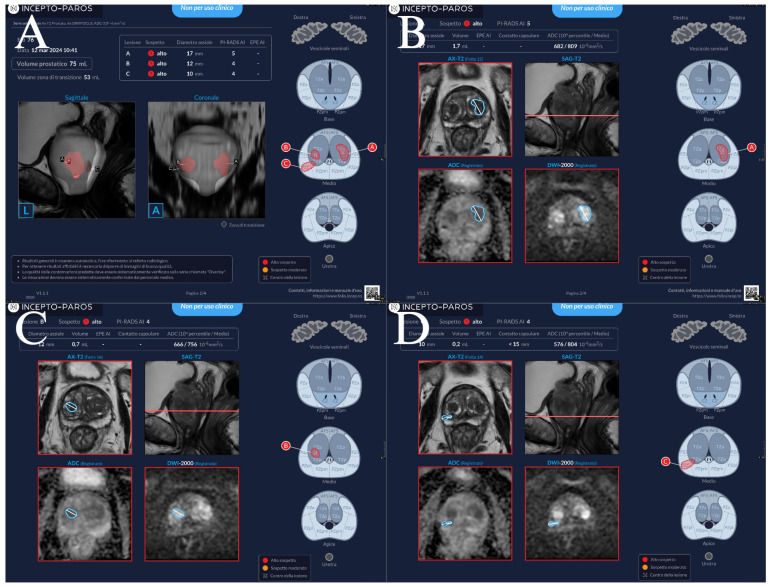
False positive prostate MRI reported by AI-powered tool (**A**). In this prostate MRI, the AI identified one PI-RADS lesion with a score of 5 in the transition zone (**B**), and two PI-RADS lesions with a score of 4 in the transition (**C**) and in the peripheral zone (**D**). The radiologist did not report any lesion with a PI-RADS score of 3 or higher. At biopsy, no prostate cancer was found. Clinical implication: this example highlights the potential risk of AI-driven overestimation of lesion suspicion, which could increase unnecessary biopsy if AI outputs are used without expert radiologist verification.

The radiologist’s sensitivity and specificity were not significantly different from those of the AI when using a PI-RADS cut-off of 3 (*p* = 0.157 and *p* = 0.346, respectively) or 4 (*p* = 1.000 and *p* = 1.000, respectively). Two examples of false-positive lesions, as reported by the radiologist and the AI, are illustrated in Figure 4 and Figure 5, respectively.

A per-lesion analysis could not be performed using biopsy results because, in cases with multiple PI-RADS ≥ 3 lesions within the same prostatic lobe identified by either the radiologist or the AI, the histopathology report did not allow for precise localization of the tumor.

### 3.4. Correlation Between Prostate Volumes Calculated by Radiologist and AI

**Figure 6:**
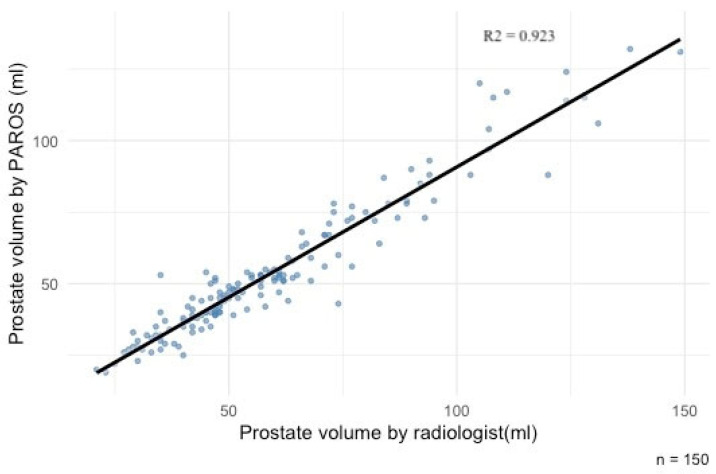
Correlation between prostate volumes (in mL) calculated by the radiologist and AI-powered PAROS. The regression line is shown as a solid line.

Prostate volumes calculated by the radiologist and the AI system were strongly correlated (r = 0.961, *p* < 0.001), as shown in Figure 6. However, the radiologist’s measurements were significantly higher (average difference: 5.6 mL, *p* < 0.001). Consequently, PSA density derived from the radiologist’s volumes was significantly lower (average difference: 0.023 ng/mL^2^, *p* < 0.001).

### 3.5. Correlation Between Dimensions of PI-RADS Lesion ≥3 Measured by Radiologist and the AI

In patients with csPCa (*n* = 95), the maximum axial diameter of PI-RADS lesions ≥3 measured by the radiologist and AI were strongly correlated (rho = 0.827, *p* < 0.001). However, the radiologist’s measurements were significantly smaller (average difference: 3 mm, *p* < 0.001).

## 4. Discussion

This study compared the detection rate of a pre-commercial trial version of PAROS, a deep learning–based AI tool, with that of an experienced radiologist in the interpretation of prostate MRI, using biopsy histopathology as the reference standard. The AI system demonstrated sensitivity and specificity comparable to the radiologist. While PLRs were modest, the extremely low NLRs observed for both PAROS and the radiologist indicate strong rule-out capability for csPCa.

These findings suggest that this AI tool may be particularly valuable in clinical scenarios where excluding csPCa is critical. Given that PLRs below 2 confer only a modest increase in post-test probability, the primary strength of PAROS lies in its role as a decision-support tool for ruling out disease rather than as a stand-alone diagnostic classifier. The exceptionally low NLR means that a negative PAROS assessment (PI-RADS < 3) substantially reduces the probability of csPCa, a feature that could be leveraged to avoid unnecessary biopsies in patients with equivocal findings.

The practical integration of PAROS into routine workflows should therefore be viewed through the lens of “assistance, not replacement,” and its utility may differ based on the clinical context and available expertise. In high-volume, specialized centers, PAROS could function as a concurrent reader, helping to flag lesions that might be overlooked and increasing confidence in concordant readings. For instance, when an experienced radiologist identifies a PI-RADS 3 lesion, a concordant PAROS score of 3 reinforces the indeterminate nature of the finding, while a discrepant PAROS score ≥ 4 may prompt a second look or increase suspicion, thereby refining risk stratification. Likewise, concordant scoring of PI-RADS ≥ 4 lesions between the radiologist and PAROS, as observed in 82.8% of cases in our study, increases the likelihood of csPCa at biopsy, supporting its potential role in guiding biopsy decisions.

Conversely, in resource-limited settings—such as low- and middle-income countries where access to radiologists with dedicated prostate MRI expertise is scarce—the role of the AI system could be more foundational. In these contexts, the tool could empower general radiologists, residents, or even trained urologists to achieve a level of diagnostic performance closer to that of an expert. The high rule-out capability (low NLR) would be particularly impactful, allowing non-specialist readers to confidently identify patients who are very unlikely to have csPCa and can thus be spared from unnecessary, costly, and often inaccessible biopsies.

In per-lesion analysis, the radiologist detected 169 lesions and the AI 227, with concordant identification in 131 (77.5%). PAROS thus identified more suspicious lesions, which may reflect greater sensitivity but also a risk of false positives, particularly in the transition zone. Nevertheless, the majority of radiologist-detected lesions were also captured by the AI, supporting its ability to recognize clinically relevant findings.

Beyond lesion detection and PI-RADS classification, a growing body of research has explored AI and radiomics approaches aimed at predicting ISUP grade or the probability of clinically significant prostate cancer before biopsy by integrating quantitative MRI features with clinical variables such as PSA, PSA density, and age. These multivariable models have demonstrated improved patient-level risk stratification and biopsy selection compared with PI-RADS assessment alone. For example, radiomics-clinical nomograms combining MRI texture features with PSA-derived metrics have shown higher accuracy for predicting csPCa or ISUP ≥ 2 disease than conventional clinical models or PI-RADS alone. Similarly, deep-learning–based MRI models have achieved robust pre-biopsy prediction of clinically significant cancer across multicenter datasets. In this context, the present findings should be viewed as complementary rather than overlapping: PAROS functions primarily as an imaging-interpretation support tool, providing standardized lesion detection and grading that could serve as structured input for downstream multivariable risk-prediction models. The very low negative likelihood ratios observed in our study reinforce the potential role of AI-assisted MRI interpretation in safely ruling out csPCa at the imaging stage, while integrated radiomics-clinical models may further refine individual biopsy decisions within established risk-stratification pathways [24,25,26].

AI in prostate MRI offers several potential benefits, including faster reporting, assistance for less experienced radiologists to reach detection accuracy similar to that of seasoned professionals, and support for second opinions. Additionally, it can help reduce inter-reader variability while also demonstrating the ability to identify and grade prostate cancer on biopsy with an accuracy rivaling that of expert histopathologists [17,21].

However, workflow integration remains a challenge: Forookhi et al. [21], reported longer reporting times with AI, mainly due to image uploading, highlighting the need for optimized PACS integration. Moreover, according to an ESR survey, robust data on AI’s impact on reducing radiologist workload are still lacking [17,27].

Since current AI systems have predominantly been trained on biparametric MRI, the widespread adoption of AI in prostate MRI could pave the way for eliminating the need for Gadolinium-based imaging. This shift would reduce healthcare costs and minimize risks to patients and environmental impact. Such a transition would be particularly beneficial in serial examinations for monitoring prostate cancer patients on active surveillance and would be essential in a screening context [28].

Despite these promising results, several challenges remain in developing and implementing AI in prostate MRI [17]. Existing datasets are often small and inconsistent in quality, limiting progress. Large, multicenter initiatives such as the PI-CAI (Prostate Imaging–Cancer AI) challenge have demonstrated the remarkable potential of AI, leveraging a large number of cases and involving multiple readers to validate its capabilities [4].

The human factor remains critical, as prostate MRI interpretation involves integrating clinical data and radiologist expertise. Radiologists are essential for ensuring AI’s clinical relevance, and collaboration with data scientists is vital for developing meaningful AI models.

From the patients’ perspective, this topic is equally intriguing. According to a recent survey of 212 patients with suspected csPCa, the majority of patients preferred AI involvement in their PCa diagnosis alongside a radiologist. However, responsibility for misdiagnosis is perceived as shared among hospitals, radiologists, and developers [29].

Concerning prostate total volume and prostate lesion measurements, PAROS evaluations showed strong correlation with those of the radiologist, potentially reducing reporting time and inter-reader variability [14]. The systematic differences observed between PAROS and the radiologist in prostate volume and lesion size measurements may have clinically relevant downstream implications, particularly for PSA density-based risk stratification. Because PSA density is inversely related to prostate volume, the smaller AI-derived volumes in our study resulted in higher PSA density estimates compared with radiologist-derived values. Given that PSA density thresholds are widely used to guide biopsy decisions—especially in patients with equivocal PI-RADS 3 lesions—such systematic shifts could move some patients across decision thresholds and influence biopsy recommendations. These findings suggest that calibration or validation of PSA density thresholds may be necessary when transitioning from manual to automated prostate volume measurements, and that integrated clinical-AI decision models should account for measurement methodology to avoid unintended changes in biopsy selection.

The patient cohort was representative of the general population in terms of lesion distribution, with peripheral zone lesions being more prevalent than the other zones [30].

An additional strength of our study was using the PI-QUAL v.2 score to exclude MRI exams of inadequate quality. However, we did not separately analyze exams with acceptable quality (PI-QUAL v.2 score of 2) versus those with optimal quality (PI-QUAL v.2 score of 3) which could provide further insight, as emerging deep-learning tools for MRI quality assessment suggest [31,32].

While this study yields interesting results, several limitations should be acknowledged. The retrospective design and relatively small sample size may restrict the generalizability of the findings.

Given the high prevalence of csPCa (96/150) and the small proportion of negative MRI examinations (16/150) in our cohort, the present results should not be extrapolated to screening or low-risk populations and instead reflect performance in a clinically enriched, high-suspicion setting. Therefore, a critical next step to validate the clinical utility of PAROS will be to conduct prospective studies in larger, more balanced cohorts that include both high-risk patients and a representative sample of low-risk individuals, including those with negative MRI findings. Such studies, ideally with long-term follow-up or using methods to account for verification bias, are essential to accurately determine the tool’s true diagnostic performance and its potential impact on reducing unnecessary biopsies in routine clinical practice, particularly in screening or low-prevalence settings.

Moreover, for this study we used a trial, non-commercial version of the software released prior to its market launch in July 2024. Since newer versions of the software have been released since its market introduction, our study may underestimate the actual detection rate achievable with the commercially available version of PAROS.

The comparison between PAROS (biparametric) and the radiologist (multiparametric) introduces a potential source of bias, as the readers had access to different input data. While the role of DCE in PI-RADS v2.1 is limited to upgrading peripheral zone PI-RADS 3 lesions, and the literature suggests biparametric and multiparametric protocols may achieve comparable diagnostic accuracy, we cannot exclude the possibility that DCE influenced the radiologist’s confidence, lesion detection, or final scoring in ways not captured by our analysis. Future studies should consider comparing AI performance against radiologist reads using both biparametric and multiparametric protocols to isolate the specific contribution of DCE when AI is used as a decision-support tool.

Our MRI protocol employed a FOCUS DWI sequence, which may enhance lesion conspicuity relative to conventional DWI. This optimized sequence could have contributed to the detection rate of both the radiologist and the AI, potentially limiting the generalizability of our findings to institutions using conventional DWI acquisitions.

A limitation of our study is the lack of stratified analysis of PAROS diagnostic performance according to prostate zone (peripheral vs. transition zone). Although most csPCa cases in our cohort were located in the peripheral zone, a substantial proportion of lesions detected exclusively by PAROS were in the transition zone. However, reliable zone-specific accuracy assessment was not feasible due to the limited number of transition zone cancers and the inability to consistently match individual MRI-detected lesions with biopsy-positive cores when multiple lesions were present within the same lobe. Future studies with precise lesion-to-biopsy spatial mapping should evaluate PAROS performance separately across prostate zones.

Finally, this was a single-center study in which all prostate MRI exams were conducted on the same 3T MRI scanner and interpreted by a single experienced radiologist with 25 years of expertise. While this ensures a high-quality reference standard for lesion detection and characterization, it also represents a significant limitation. The absence of multiple readers with varying levels of experience prevents us from drawing any conclusions about PAROS’s potential impact on supporting less experienced readers to achieve expert-level performance. To address this question, future prospective studies should adopt a multi-reader, multi-center design that includes radiologists with different levels of experience (from novice to expert). Such studies are essential to rigorously evaluate PAROS’s true value as a decision-support tool and its ability to standardize prostate MRI reporting in real-world clinical settings.

## 5. Conclusions

This study demonstrates that the evaluated AI-powered tool achieves a detection rate for clinically significant prostate cancer on MRI comparable to that of an experienced radiologist. However, the retrospective, single-center design, the use of a single expert reader, and the limited sample size highlight the need for larger, prospective, multi-center studies including less-experienced readers to validate these findings.

## Figures and Tables

**Table 1:** Characteristics of the 150 patients.

Median Age in Years (IQR)	67 (61.25–72)
Median total PSA in ng/mL (IQR)	6.1 (4.7–9.7)
Median RVol in ml (IQR)	52 (42–71)
Median PSA density in ng/mL (IQR) derived by RVol	0.119 (0.075–0.192)
Median PVol in ml (IQR)	48 (39–63.7)
Median PSA density in ng/mL (IQR) derived PVol	0.131 (0.083–0.218)
No Pca at biopsy, patients	41 (27.3%)
Gleason score of 6, patients	14 (9.3%)
CsPca at biopsy, patients	95 (63.3%)
Gleason score of 7	66 (44%)
Gleason score of 8	23 (15.3%)
Gleason score of 9	6 (4%)
csPCa in peripheral zone, patients	68 (71.6% of csPCa)
csPCa in transition zone, patients	8 (8.4% of csPCa)
csPCa in anterior fibromuscular stroma (AFS), patients	3 (3.1% of csPCa)
csPCa in central zone, patients	1 (1% of csPCa)
csPCa extending in two or more zones, patients	15 (15.8% of csPCa)
Median diameter in mm of csPCa measured by radiologist (IQR) *	14 (11–22)
Median diameter in mm of csPCa measured by PAROS (IQR) *	18 (12–27)

csPCa: clinically significant prostate cancer; IQR: interquartile range; PVol: prostate volume calculated by AI-powered PAROS; RVol: prostate volume with ellipsoid formula calculated by radiologist; * The diameter refers to the PI-RADS lesions with the highest score.

**Table 2:** Detection rate with 95% C.I. of radiologist and the AI software across different PI-RADS category cut-offs.

		RADIOLOGIST	AI
PI-RADS ≥ 3	Sensitivity	100% (96–100)	97.9% (93–100)
	Specificity	29.1% (18–44)	21.8% (12–36)
	PLR	1.41 (1.20–1.69)	1.25 (1.09–1.46)
	NLR	0 (0–0)	0.096 (0.022–0.40)
PI-RADS ≥ 4	Sensitivity	95.8% (91.8–99.8)	95.8% (91.8–99.8)
	Specificity	49.1% (35.8–62.4)	49.1% (35.8–62.4)
	PLR	1.88 (1.44–2.46)	1.88 (1.44–2.46)
	NLR	0.085 (0.031–0.231)	0.085 (0.031–0.231)

NLR: negative likelihood ratio; PLR: positive likelihood ratio.

## Data Availability

The raw data supporting the conclusions of this article will be made available by the authors on request.

## Supplementary Materials

The following supporting information can be downloaded at https://www.mdpi.com/article/10.3390/curroncol33030151/s1, Table S1: Compounds analysed in the 2020 moss survey.

## Abbreviations

The following abbreviations are used in this manuscript:

ADC	apparent diffusion coefficient
AI	Artificial intelligence
AUC	Area under the curve
csPCa	clinically significant prostate cancer
DWI	diffusion-weighted imaging
EAU	European Association of Urology
ESUI	European Society of Urological Imaging
ESUR	European Society of Urogenital Radiology
mpMRI	multi parametric MRI
PCa	prostate cancer
PI-RADS	Prostate Imaging Reporting and Data System
PI-QUAL	Prostate Imaging Quality
SROC	summary receiver operating characteristic
TURP	transurethral resection of prostate
