# Supplementary material for: Artificial Intelligence in Prostate MRI: Comparison of an AI-Based Software and an Experienced Radiologist for Detecting Clinically Significant Prostate Cancer
Source: Curr Oncol. 2026 Mar 6;33(3):151. doi: 10.3390/curroncol33030151 (PMC13025867; doi:10.3390/curroncol33030151)
Supplement: Supplementary file 1 [file curroncol-33-00151-s001.zip › curroncol-4130716-supplementary.pdf]

Supplementary Material

Supplementary Table S1. Acquisition parameters for Prostate mpMRI.

	T2WI	FOCUS DWI	DCE
Sequence	Fast Recovery Fast Spin Echo (FRFSE)	Echo planar imaging (EPI)	DIfferential Subsampling with Cartesian Ordering (DISCO)
Acquisition plane	Axial, Sagittal and Coronal	Axial	Axial
In-plane resolution (mm x mm)	0.48 (frequency) x 0.38 (phase)	2 (frequency) x 1.78 (phase)	1.2 (frequency) x 1.2 (phase)
Slice thickness	3	3	3
Matrix size	420 x 332	110 x 56	172 x 160
Field of view	16 x 16 cm	FOCUS 22 x 10 cm	20 x 20 cm
B values	n/a	50-800-1000-2000	n/a
Temporal resolution (sec)	n/a	n/a	7
Contrast media	n/a	n/a	Gadobutrol/Gadoteridol (0.1 mmol/kg at 2 cc/s)
